# Cumulative adverse childhood experiences and their association with depression and anxiety: a cross-sectional study of youth living in informal urban settings in Kenya

**DOI:** 10.3389/fpsyt.2025.1641321

**Published:** 2025-09-10

**Authors:** William Byansi, Catherine Mawia Musyoka, Christopher E. Baidoo, Moses Okumu, Teresia Mutavi, Anne Mbwayo, Angeline Sabina Mulwa, Dorothy Ndunge Kyalo, Muthoni Mathai

**Affiliations:** ^1^ Boston College, School of Social Work, Chestnut Hill, MA, United States; ^2^ Department of Psychiatry, Faculty of Health Sciences, University of Nairobi, Nairobi, Kenya; ^3^ School of Social Work, University of Illinois Urbana-Champaign, Urbana, IL, United States; ^4^ Department of Educational Management, Policy and Curriculum Studies, Faculty of Education, University of Nairobi, Nairobi, Kenya

**Keywords:** adverse childhood experiences, depression, anxiety, stress, urban slums, informal environments

## Abstract

Adverse childhood experiences (ACEs), such as abuse, neglect, and household dysfunction, are linked to adverse mental health outcomes, particularly among adolescents and young adults living in informal urban settlements, where poverty, unemployment, and exposure to violence exacerbate early life adversity. Despite this, research on ACEs and mental health in these contexts remains limited, especially among youth living in informal urban settlements in Nairobi, Kenya. Between September and December 2024, we conducted a community-based study involving 94 youth aged 15 – 24 years living in two informal settlements in Nairobi. Participants were recruited through peer-driven sampling if they had used alcohol or drugs in the past 30 days prior to the study. Data were collected using tablet-based surveys administered in English or Swahili on mental health outcomes of depression (using PHQ - 9), anxiety (assessed by GAD - 7), stress (assessed by stress scale), and ACEs (evaluated by the ACEs scale). We conducted generalized linear models to examine the relationship between ACEs (0 – 2 vs. 3+ experiences) and mental health outcomes. The median age was 21. Most participants were male (54%) and reported three or more ACEs (56%). Even though depression and anxiety scores were low, youth with 3+ ACEs had significantly higher depression and anxiety scores than those with fewer ACEs, but no significant association with stress symptoms was observed. There is a need for early identification of ACEs and the integration of psychosocial support into community-based youth services to prevent the long-term effects of childhood adversity.

## Introduction

Mental health challenges represent a disproportionately high burden among youth in Africa, with 40.8% experiencing emotional and behavioral problems, 29.8% suffering from anxiety disorders, 26.9% facing depression, 24% exhibiting post-traumatic stress disorder (PTSD), and 20.8% reporting suicidal ideation (1). This burden is even higher among adolescents living in informal urban settlements in Kenya due to the intersecting realities of poverty, overcrowding, violence, breakdown of social networks, and limited access to services (2–5). For instance, recent findings have found that adolescent girls exposed to gender-based violence in informal settlements reported elevated levels of depression (9.2%), anxiety (17.6%), and post-traumatic stress disorder (12.2%) (6). Similarly, a study among Kenyan high school students revealed that 45.9% experienced depression and 38.0% faced anxiety, and these conditions were significantly influenced by gender and social support (7). To inform tailored interventions, there is a need to examine how adverse childhood experiences (ACEs), such as abuse, neglect, and household dysfunction, contribute to the etiology of mental health symptoms among youth in informal settlements.

ACEs are traumatic events occurring before the age of 18 years, including abuse, neglect, and household challenges (8). ACEs often cluster in marginalized urban settings and serve as predictors and compounding factors for poor mental health outcomes (9–11), making them a crucial focus for prevention and intervention efforts targeting vulnerable groups. Psychological stress resulting from ACEs significantly changes how children develop, leading to lasting effects on their health, behaviors, and life potential in adulthood (12). Research in Africa has consistently linked ACEs to chronic physical and mental health problems such as PTSD, substance use disorders, depression, injuries, and adverse outcomes for mothers and children (13, 14). Moreover, ACEs are associated with lower educational attainment, unemployment, and reduced earning potential (15). Widespread exposure to ACEs is evident in population-level data across Africa. For example, a recent multi-country analysis of the Violence Against Children and Youth Surveys found that 55.0% of men and 37.2% of women have witnessed physical violence and that 49.7% of males and 36.5% of females have directly experienced it (9). In many seminal studies, ACEs are routinely measured using a binary classification, wherein the presence or absence of each adversity is recorded and summed up to produce an overall ACE score (16). This cumulative score is frequently categorized into ranges, such as 0, 1, 2 – 3, and ≥ 4 ACEs (16). However, this reductionist approach has certain limitations, including insufficient consideration of the specific nature or severity of different adversities (17). As a result, it masks heterogeneity in risk and may undermine the nuanced understanding necessary for targeted intervention and policy response.

A life-course epidemiological perspective provides a framework for understanding how childhood experiences and exposures may contribute to long-term health trajectories, particularly through cumulative adversity (17). Adolescents living in informal urban settlements face layered vulnerabilities that place them at high risk for mental health challenges (10) and could have long-term effects. Nevertheless, mental health research focusing on this group remains sparse, especially for youth residing in Nairobi’s urban informal settings, where structural inequities and limited access to supportive services exacerbate the burden of ACEs and mental health distress in this population. While global evidence links ACEs to poor mental health outcomes, there is limited evidence on how ACEs may shape youth mental health difficulties, particularly in informal urban settings such as Kibera. Guided by the life course perspective, this study fills the knowledge gap by examining the association between ACEs and symptoms of depression, anxiety, and stress among youth aged 15 – 24 years living in Nairobi’s informal settlements. We hypothesized that youth with three or more adverse childhood experiences would have significantly higher depression, anxiety, and stress symptom scores than those with fewer ACEs. The findings of this study can inform targeted mental health interventions and policy efforts tailored to the unique experiences of youth in high-adversity environments.

## Methods

### Sample

This study utilized baseline data from a pilot cluster-randomized mixed-methods sequential study conducted in two informal settlements in Nairobi, Kenya. One settlement was assigned to the control arm, and the other was assigned to the treatment arm. The main goal of the parent study was to assess an intervention designed to reduce alcohol and substance use among youth in informal urban settings. In this brief report, we used baseline data before the participants received any intervention and were unaware of their group assignment. The study was conducted in Mukuru Kwa Reuben and Kibra, two large informal settlements in Nairobi that are home to vibrant and diverse communities (18). Both areas are characterized by high population density and limited infrastructure, with families often living in small one-room dwellings and sharing communal water points and sanitation facilities (19). Despite these challenges, residents demonstrate strong social networks and resilience. At the same time, youth face heightened risks related to unemployment, limited access to health and social services, exposure to substance use and violence, making these settings highly relevant for research on adolescent well-being (19, 20).

Data were collected from a community sample between September and December 2024. A snowball sampling method was employed to recruit 94 youth aged 15 – 24 years. The initial recruitment team, consisting of two boys and one girl serving as community health promoters (CHPs), identified peers who were known to use alcohol and other drugs. These CHPs then referred additional youth for participation. Participants aged 15 – 17 years were typically in high school, while those aged 18 – 24 years were mostly in college. However, several individuals across both age groups were not enrolled in school due to economic hardship or engagement in delinquent behavior, both of which are common in informal settlements. Both in-school and out-of-school youth were eligible for this study. Youth were excluded if they were outside the 15 – 24 age range or exhibited severe substance use or mental health symptoms that required inpatient treatment.

### Procedures

Five research assistants (RAs) were recruited through a competitive hiring process after a public call for applications. Candidates were assessed for their prior research experience and interpersonal skills during interviews. Selected RAs completed an online certification in Good Clinical Practice administered by the NIDA Clinical Trials Network. Participant recruitment was conducted in collaboration with two local youth-serving organizations in Mukuru Kwa Reuben and Kibera, Nairobi, Kenya. RAs and community health promoters distributed flyers outlining the study details, including the location and timing. Written informed consent or assent was obtained from all participants and parental or caregiver consent for those under 18 years, with separate procedures to avoid coercion. Eligibility screening was conducted in person, and interviews were conducted by trained RAs fluent in English and Swahili. The surveys were interviewer-administered in the participant’s preferred language and lasted approximately 30 minutes. To protect privacy, all the interviews were conducted in private settings. Participants received 500 Kenyan Shillings (approximately $5) as compensation for their time. The study was approved by the Kenyatta National Hospital and the University of Nairobi Ethics Review Committee (KNH-UoN ERC No. P423/05/2024), and the National Commission for Science, Technology, and Innovation (NACOSTI/P/24/39331).

### Measures

Independent variable: Adverse Childhood Experiences was assessed using the 10-item Adverse Childhood Experiences–International Questionnaire (21). The questionnaire included items on emotional and/or physical neglect; emotional, sexual, and/or physical abuse; bullying; cohabitation with someone with a substance use disorder and/or a mental disorder; having an incarcerated household member; witnessing domestic violence; experiencing parental loss and/or divorce/separation; witnessing community violence; and exposure to collective violence (e.g., war, terrorism, and political violence). Each item was treated as a binary variable (1 = experienced, 0 = not experienced). A cumulative ACE score was calculated by summing the responses across all ten items, and based on existing literature (22), scores were then categorized into two groups, 0 – 2 and 3 or more ACEs (Cronbach’s alpha = 0.85).

Outcome variables: The study examined three dependent variables: depressive symptoms, anxiety symptoms, and stress. *Depressive symptom severity* in this study was evaluated using the 9-item Patient Health Questionnaire (23). This scale employs a 4-point Likert-type response format, ranging from 0 (“not at all”) to 3 (“nearly every day”). This scale has been widely used and validated in many African settings (24). Consistent with standard practice, we used the scale as a continuous outcome, with higher scores indicating greater depressive symptoms (24–26). The measure demonstrated strong internal consistency (Cronbach’s α = 0.80).

*Anxiety symptoms* were evaluated using the 7-item Generalized Anxiety Disorder Scale (GAD - 7) to measure anxiety symptoms (27). This validated measure, suitable for clinical and research use across diverse cultural contexts, employs a Likert-type scale to assess the severity of anxiety (0 = not at all to 3 = nearly every day). Participants reported on their experiences over the preceding two weeks, responding to items such as “feeling nervous, anxious, or on edge” and “not being able to stop or control worrying.” The total score ranges from 0 to 21, with higher scores indicating greater anxiety symptoms (27). The GAD - 7 demonstrated acceptable internal consistency in the current study (Cronbach’s α = 0.81).

*Stress* was measured using a 10-item Perceived Stress Scale (PSS) (28). Participants were asked to evaluate their stressful life experiences in the past month. Possible summated scores ranged from 0 to 40, with higher scores indicating greater symptom severity. In this study, the scale demonstrated acceptable internal consistency (Cronbach’s α = 0.79).

Control variables: We controlled for participants’ sociodemographic factors of age (continuous), gender (categorical: male and female), employment status (categorical: working vs. not working), education (categorical: secondary or less vs. higher than secondary), and living situation (categorical: living with a caregiver vs. independent living).

### Statistical analysis

We performed descriptive statistics and bivariate tests of association appropriate for each variable’s level of measurement. We estimated generalized linear models, which are not subject to ordinary least squares regression assumptions. To inform model specification, we assessed the distribution of each outcome using histograms and descriptive statistics. Stress was approximately normally distributed, whereas depression and anxiety were positively skewed. Based on these assessments, we specified a Gaussian distribution for the stress outcome and gamma distributions for depression and anxiety. Because Gamma models require positive outcomes and some depression and anxiety scores were zero, we added a 0.01 constant to all depression and anxiety scores. All models used log links, and the coefficients were exponentiated to aid interpretability. Analyses were conducted using Stata Now 19.5 (StataCorp, College Station, TX).

## Results

### Participant characteristics

The median age of the 94 participants was 21 years. Most participants were male (n=51; 54%), had secondary education or less (n=76; 81%), were not working (n=60; 64%), and lived with a parent or guardian (n=66; 70%). Bivariate analyses—unadjusted for other factors—revealed that participants with 3+ ACES had median depression scores that were significantly higher than those with 0 – 2 ACES, and those with higher than secondary education reported significantly greater stress symptoms than participants with secondary education or less (**Table 1**).

**Table 1:** Descriptive statistics (N=94).

Variables	N (%) or Median (IQR)	Stress		Depression		Anxiety	
		Mean (SD)	p-value	Median (IQR)	p-value	Median (IQR)	p-value
Total Sample	94	19.1 (7.5)		7 (7)		5 (8)	
Age, years	21 (4)	–	0.273	–	0.247	–	0.950
Age			0.128		0.360		0.902
15-17	16 (17.0)	17.3 (6.2)		4.5 (6.0)		5.0 (5.0)	
18-19	24 (25.5)	17.4 (7.8)		6.5 (8.5)		5.0 (7.5)	
20-22	31 (33.0)	20.8 (7.2)		7.0 (6.0)		5.0 (7.0)	
23-25	23 (24.5)	19.7 (8.3)		9.0 (9.0)		6.0 (8.0)	
Sex			0.715		0.058		0.049
Female	43 (45.7)	19.6 (7.3)		8.0 (8.0)		6.0 (7.0)	
Male	51 (54.3)	18.7 (7.7)		6.0 (7.0)		5.0 (6.0)	
Education			0.007		0.291		0.191
Secondary or less	76 (80.9)	18.2 (7.4)		8.0 (7.0)		5.5 (8.0)	
Higher than secondary	18 (19.2)	22.9 (6.9)		6.0 (5.0)		4.0 (4.0)	
Employment			0.956		0.816		0.972
Not working	60 (63.8)	19.2 (7.5)		6.5 (7.0)		5.0 (7.5)	
Working	34 (36.2)	18.8 (7.7)		7.5 (6.0)		5.5 (7.0)	
Living situation			0.568		0.624		0.567
With parent/guardian	66 (70.2)	19.0 (7.1)		7.0 (7.0)		5.0 (8.0)	
Alone or with spouse	28 (29.8)	19.3 (8.6)		7.0 (7.5)		5.0 (7.0)	
ACEs			0.153		0.000		0.003
0-2	40 (42.6)	18.1 (7.7)		4.0 (6.5)		4.0 (3.5)	
3+	54 (57.5)	19.9 (7.4)		9.0 (7.0)		6.5 (6.0)	

SD, standard deviation; IQR, interquartile range.

p-values were derived from Spearman correlations for continuous age, Kruskal-Wallis tests for categorical age, and Wilcoxon rank-sum tests for binary variables.

Stress was measured on a 0 – 40 scale, depression on a 0 – 27 scale, and anxiety on a 0 – 21 scale. Higher scores indicate greater symptom severity.

### Cumulative ACEs, depressive, anxiety, and stress symptoms

GLM analyses, which adjusted for all covariates, showed that youth with 3+ACEs had 61% (exp(β)=1.61, 95% CI: 1.16, 2.26, *p*<.01) higher depression scores and 64% (exp(β)=1.64, 95% CI: 1.19, 2.28, *p*<.01) higher anxiety scores than those with fewer ACEs, with no significant association with stress symptoms (**Table 2**). Participants with higher education reported significantly greater stress symptoms than those with secondary or less education (exp(β)=1.30, 95% CI: 1.06, 1.60, *p*<.05). Males reported significantly lower anxiety levels than females (exp(β)=.69, 95% CI: 0.48, 0.99, *p*<.05).

**Table 2:** GLM regression results (N=94).

Variables	Stress Model exp(β) (95% CI)	Depression Model exp(β) (95% CI)	Anxiety Model exp(β) (95% CI)
ACEs			
0-2	Referent	Referent	Referent
3+	1.11 (0.94, 1.31)	1.61 (1.16, 2.26)**	1.64 (1.19, 2.28)**
Age	1.00 (0.96, 1.04)	1.00 (0.93, 1.08)	0.96 (0.89, 1.03)
Sex			
Female	Referent	Referent	Referent
Male	0.95 (0.79, 1.14)	0.76 (0.53, 1.09)	0.69 (0.48, 0.99)*
Education			
Secondary or less	Referent	Referent	Referent
Higher than secondary	1.30 (1.06, 1.60)*	0.85 (0.55, 1.32)	0.87 (0.57, 1.33)
Employment			
Not working	Referent	Referent	Referent
Working	1.04 (0.83, 1.31)	1.01 (0.67, 1.54)	1.27 (0.83, 1.94)
Residential			
Living with a parent/guardian	Referent	Referent	Referent
Living alone or with a spouse	0.96 (0.78, 1.17)	1.04 (0.67, 1.59)	1.04 (0.69, 1.59)

*p<.05, **p<.01.

## Discussion

Our findings indicate that youth in informal urban settlements who reported three or more adverse childhood experiences (ACEs) had significantly greater symptoms of depression and anxiety than those with fewer ACEs. This pattern supports the life course model, emphasizing how cumulative early adversity, common in high-risk environments such as informal urban environments, can disrupt mental health well into adolescence and young adulthood. While ACEs were not significantly linked to stress symptoms in this study, the strong associations with both depression and anxiety underscore the critical need for trauma-informed mental health services that consider early life experiences. The median depression and anxiety scores in our sample fell below the established clinical cut-offs for probable major depression (≥10 on the PHQ - 9) and generalized anxiety disorder (≥10 on the GAD - 7). This suggests that our participants may represent a non-clinical population. Nonetheless, even sub-clinical symptom elevations are relevant in high-adversity settings, as they may progress to clinically significant disorders if left unaddressed.

ACEs have been linked to adverse mental health outcomes, including depression, anxiety, and PTSD, in many African settings (13, 14, 29) and resource-constrained settings in the Western World (8, 12). Although ACEs impact everyone, their effects on youth in informal settlements are amplified by the structural and social conditions that shape their health trajectories (10). For instance, in Kenya, intersecting structural issues of unemployment, violence, unstable housing, poverty, and inadequate sanitation facilities converge in informal urban settlements and exacerbate the impact of ACEs on overall youth well-being, negatively impacting mental and physical health outcomes (6, 10, 20). Additionally, the high prevalence of ACEs may reflect the cumulative psychosocial stressors that youths face in informal urban settlements, including violence, caregiving responsibilities, lack of educational opportunities, and limited mobility (7, 30, 31). The results emphasize the importance of contextual interventions that address the structural and interpersonal challenges that youths encounter across developmental stages in high-adversity contexts.

In the context of informal urban settlements, where educational attainment is often hard-won amidst adversity, increased academic and economic pressures among more educated youth may increase mental symptom severity. Youth in informal urban settlements may also encounter heightened expectations or uncertainty regarding future opportunities, contributing to elevated symptoms of depression, anxiety and stress. Therefore, we argue that while education is often protective, it can also serve as a source of psychological burden in structurally disadvantaged environments (14, 32, 33). Additionally, in informal settlements, long travel distances to school, often requiring unsafe routes or costly transport, may contribute to higher stress levels among students, particularly those striving to attain a higher education. These logistical and financial burdens can exacerbate the psychological burden on youth who are already facing multiple adversities. On the other hand, many youths in informal urban environments may lack access to quality educational opportunities or may travel long distances to reach schools. However, many schools offer opportunities to nurture and support youth who face adversity. Therefore, it is crucial to strengthen community and family support systems for youth in informal urban settlements, as this is vital for those who might otherwise fall behind in school.

Gender differences in mental health emerged as a key theme in our analysis, with male participants reporting significantly lower anxiety symptoms than their female counterparts. These disparities may reflect both socialized gender roles and the cumulative psychosocial stressors that girls face in informal settlements, including gender-based violence, caregiving responsibilities, and limited mobility (7). Such stressors likely contribute to higher anxiety levels among female youth. This aligns with other studies indicating that gender significantly influences mental health outcomes among youth in low-resource settings (34–36). For example, Rescorla and colleagues (34) analyzed gender differences in mental health symptoms across 31 societies (n = 55,508), which included both developed and developing countries. The study revealed that female adolescents experienced greater overall emotional distress and reported more depressive symptoms than their male counterparts. The results emphasize the importance of gender-sensitive interventions that address both the structural and interpersonal challenges female youths encounter across developmental stages in high-adversity contexts.

This study had several limitations that should be considered when interpreting the findings. First, the small sample size (N = 94) limits the generalizability of the results and decreases the statistical power to detect more nuanced associations. Second, the cross-sectional design prevented us from establishing causal relationships between ACEs and mental health outcomes, as exposures and outcomes were measured simultaneously. Although males reported significantly lower anxiety scores than females, this pilot study is not designed to explore gender differences. Several scholars caution against over interpreting control variable effects when they are not central to the study’s aims (37). Finally, all data were based on self-reports, which may be subject to recall or social desirability bias, particularly regarding sensitive topics such as mental health and ACEs.

These findings have important implications for adolescent mental health interventions in Kenya, particularly within urban informal settlements, where structural disadvantages, poverty, and social instability are pervasive. The findings underscore the critical need to strengthen adolescent mental health programming in Kenya’s informal urban settlements through early screening, targeted interventions, and task-shifted service delivery approaches (38–40). The strong association between cumulative ACEs and symptoms of depression and anxiety highlights the urgent need to implement routine screening to identify at-risk youth before symptoms become severe. Interventions should be trauma-informed, recognizing the widespread effects of early adversity and tailored to address the specific stressors present in informal urban environments. Task-shifting strategies, such as training lay counselors, teachers, or community health workers, can expand the reach of mental health services and offer culturally appropriate and scalable support (38). Collectively, these implications highlight the need for a multi-layered, equity-driven approach that integrates trauma-informed care with community-based delivery to disrupt the cycle of adversity and improve mental health outcomes for vulnerable urban youth in Kenya.

## Data Availability

The raw data supporting the conclusions of this article will be made available by the authors, without undue reservation.
